# Measurement techniques for assessing the olfactory impact of municipal sewage treatment plants

**DOI:** 10.1007/s10661-015-5024-2

**Published:** 2015-12-15

**Authors:** Jacek Gebicki, Hubert Byliński, Jacek Namieśnik

**Affiliations:** Department of Chemical and Process Engineering, Chemical Faculty, Gdansk University of Technology, Narutowicza 11/12 Street, 80-233 Gdansk, Poland; Department of Analytical Chemistry, Chemical Faculty, Gdansk University of Technology, Narutowicza 11/12 Street, 80-233 Gdansk, Poland

**Keywords:** Sewage treatment plants, Measurement techniques, Electronic nose, Olfactometry

## Abstract

The study presents information about the measurement techniques used for the assessment of air quality in terms of the olfactory intensity resulting from the operation of municipal sewage treatment plants. Advantages and disadvantages of the measurement techniques used are presented. Sources of malodourous substance emission from sewage treatment plants were described, and the malodourous substances emitted were characterised. Trends in development of analysis and monitoring of the malodourous substances in the air were also presented.

## Introduction

More and more attention is devoted to environmental protection issues, including atmospheric air quality, as a result of the introduction of the principles of green chemistry into the technological practice and other types of human activity, which result from the sustainable development concept. Dynamic economic development contributes to an increased amount of pollutants emitted into the environment, which can have a negative effect on the abiotic part of the environment as well as on living organisms, human health and life. Municipal sewage treatment plants belong to the symptoms of human pressure, which can be particularly onerous (Gostelow et al. 2001; Henshaw et al. 2006; Nicell 2009; Baltrenas et al. 2013).

In a majority of currently operating treatment plants, the sewage treatment process consists of the following stages:

- Mechanical treatment—separation of all solids, floating bodies, fats and oils
- Biological treatment—processes of contaminant decomposition, which usually occur under aerobic conditions owing to microorganisms
- Biological treatment with the removal of nutrients, i.e. nitrogen and phosphorus compounds—removal of contaminants by adding various coagulants, which facilitate the nutrient precipitation process
- Water renewal—use of various processes to improve water quality, such as filtration, coagulation, osmosis and ion exchange

**Fig. 1:**
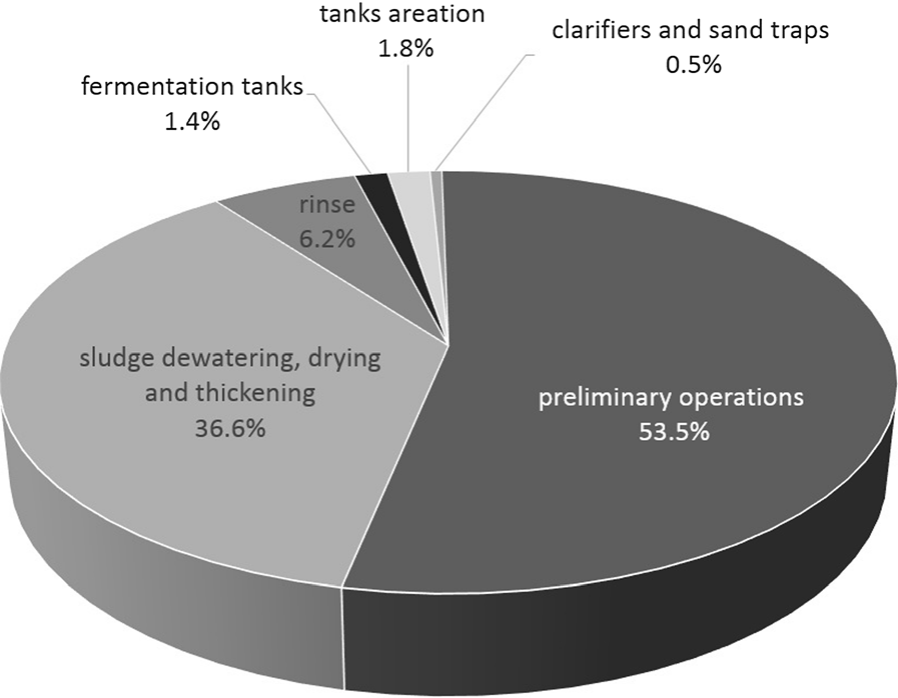
Average percentage distribution of odour emission sources from a sewage treatment plant

Amongst the sewage treatment stages listed, those related to preliminary treatment, which takes place with the use of various kinds of grilles, sand separators or initial settling tanks, where anaerobic processes occur, contribute the most to emission of malodourous substances (Stuetz and Frechen 2001). Air pollutant emissions from individual sources from the sewage treatment plant, which can influence the occurrence of malodour in adjacent areas, are presented in Fig. 1 (Naddeo et al. 2012; Lazarova et al. 2013).

Organic odourous compounds include, amongst other things, organic sulphur compounds, indoles, skatoles, organic acids, aldehydes and ketones. Table 1 summarises the information about olfactory sensations associated with some chemical compounds emitted from the sewage treatment plant (Gostelow et al. 2001).

**Table 1:** Olfactory substances emitted from a sewage treatment plant

Class of compounds	Name	Molecular formula	Type of odour
Aldehydes and ketones	Formaldehyde	HCHO	Pungent, stifling
	Acetaldehyde	CH_3_CHO	Fruity, apple
	Butyraldehyde	C_3_H_7_CHO	Rancid, odour of sweat
	Isobutyl aldehyde	(CH_3_)_2_CHCHO	Fruity
	Isovaleric aldehyde	(CH_3_)_2_CHCH_2_CHO	Fruity, apple
	Acetone	CH_3_COCH_3_	Fruity, sweet
	Butanone	C_2_H_5_COCH_3_	Apple
Carboxylic acids	Acetic acid	CH_3_COOH	Vinegar
	Butanoic acid	C_3_H_7_COOH	Rancid, odour of sweat
	*n*-Pentanoic acid	C_4_H_9_COOH	Odour of sweat
Nitrogen compounds	Ammonia	NH_3_	Sharp, pungent
	Methylamine	CH_3_NH_2_	Fish
	Dimethylamine	(CH_3_)_2_NH	Fish
	Trimethylamine	(CH_3_)_3_N	Fish, pungent
	Ethylamine	C_2_H_5_NH_2_	Pungent
	Ethylenediamine	NH_2_(CH_2_)_5_NH_2_	Rotten meat
	Pyridine	C_6_H_5_N	Unpleasant, irritant
	Indole	C_8_H_6_NH	Odour of faeces, mucilaginous
	Skatole	C_9_H_8_NH	Odour of faeces, mucilaginous
Sulphur compounds	Hydrogen sulphide	H_2_S	Rotten eggs
	Dimethyl sulphide	(CH_3_)_2_S	Rotten vegetables, garlic
	Diethyl sulphide	(C_2_H_5_)_2_S	Mucilaginous
	Diphenyl sulphide	(C_6_H_5_)_2_S	Burnt rubber
	Allyl sulphide	(CH_2_CHCH_2_)_2_S	Garlic
	Carbon disulphide	CS_2_	Rotten vegetables
	Dimethyl disulphide	(CH_3_)_2_S_2_	Rotten eggs
	Methanethiol	CH_3_SH	Rotten cabbage, garlic
	Ethanethiol	C_2_H_5_SH	Rotten cabbage
	Propanethiol	C_3_H_7_SH	Unpleasant
	Butyl mercaptan	C_4_H_9_SH	Unpleasant
	*Tert*-butyl mercaptan	(CH_3_)_3_CSH	Unpleasant
	Allyl mercaptan	CH_2_CHCH_2_SH	Garlic
	Crotyl mercaptan	CH_3_CHCHCH_2_SH	Rancid
	Benzyl mercaptan	C_6_H_5_CH_2_SH	Rancid
	Thiocresol	CH_3_C_6_H_4_SH	Rancid
		C_6_H_4_SH	
	Thiophenol	C_6_H_5_SH	
	Sulphur dioxide	SO_2_	Rotten vegetables, mucilaginous

As shown by research results described in the literature, the odours emitted from sewage treatment plants are not carcinogenic; however, their presence in the air often has a disadvantageous effect on people inducing such symptoms as headache and dizziness, malaise, concentration problems or other health hazards (Luginaah et al. 2000; Fransses et al. 2002; Rosenkranz and Cunningham 2003; Guillot 2012; Capelli et al. 2012). Moreover, their emission has a negative effect on the plant and animal ecosystem (Nicell 2009). The level of emission of these compounds into the environment is variable, and it largely depends on the sewage quality, rate of biological changes occurring in the collected sewage or technological solutions employed at the sewage treatment plants.

In the E.U., stricter and stricter legislative regulations are being adopted, which contain recommendations regarding levels of odour emissions from various sources. Special attention is paid to the issue of measuring concentrations and determining features (categories) of emitted odourants. The determination of individual odourants in atmospheric air is necessary to determine the condition of the environment in a comprehensive manner (Belgiorno et al. 2012). Despite measurements of concentrations of emitted odours, it is often required that the route of pollution dissemination should be traced and socio-technical treatment undertaken should be assessed (treatment of gas-emitting streams, air-tight sealing of devices and installations). Appropriate tools are used for measuring and controlling the level of air pollution by various xenobiotics (de Nevers 2010).

### Measurement techniques used for the assessment of atmospheric air quality in terms of odour intensity

Appropriate measurement techniques should be used to determine the level of various odourants emitted into the environment during sewage treatment plant operation or only from its individual subunits. Selection of the technique very often results from a range of factors, which, to a lesser or greater extent, may influence the final test results (variability of sample collection conditions or variability of its composition in time). It is worth emphasising that there is not one commonly accepted technique, which would allow for the effective estimation of the influence of odourous compounds on the environment (Naddeo et al. 2012).

**Fig. 2:**
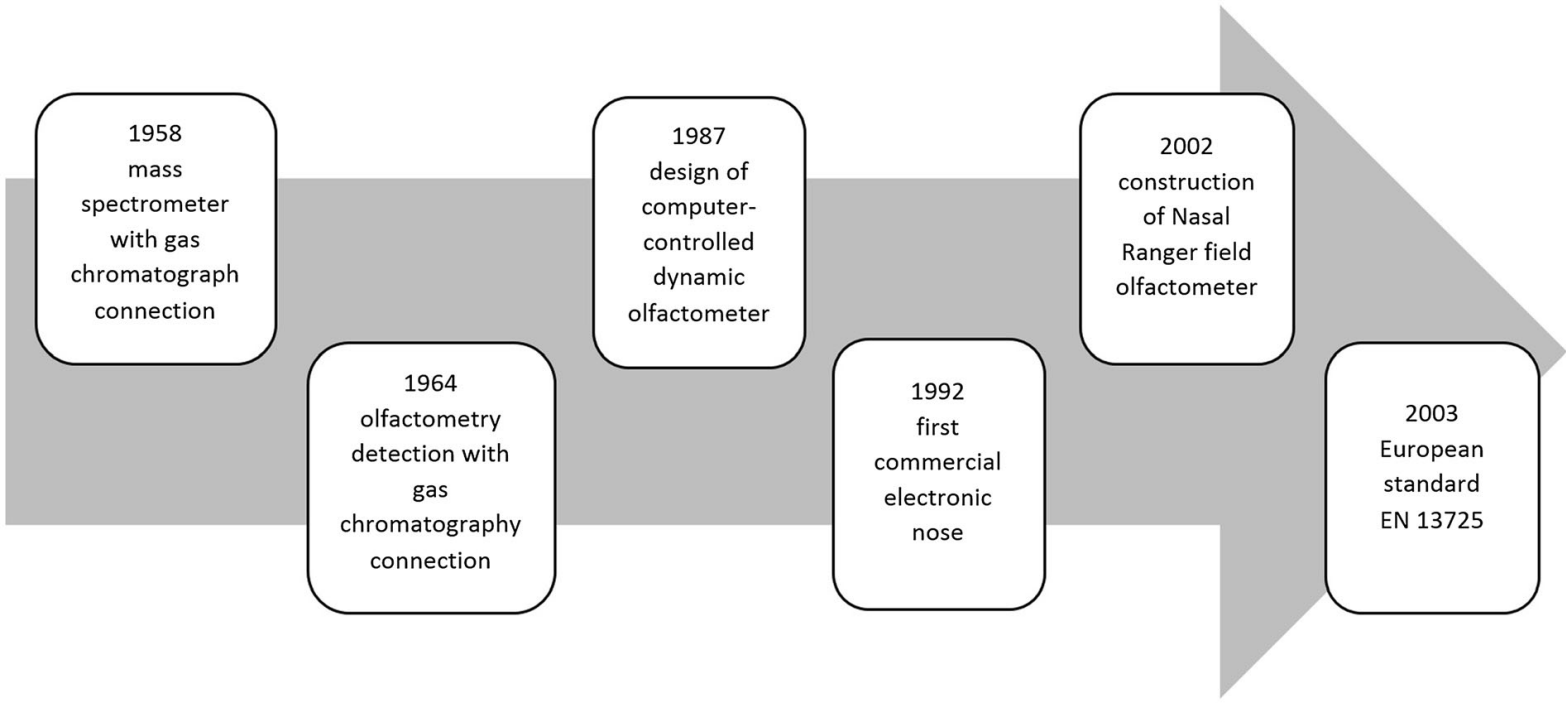
Milestones in the development of odour intensity measurement techniques

Over the past few decades, a range of new technical and processing solutions have appeared, which have had an enormous influence on the development of research in the area of measurement of atmospheric air pollution. This progress was also related to legal aspects defining the method of conducting such research. Figure 2 presents the so-called milestones regarding the development of knowledge and technology of odour intensity measurements and development of legislation pertaining to olfactory measurements (Gohlke and McLafferty 1993; Gardner and Bartlett 1994; van Ruth 2001).

Considering the most general classification of techniques used in the assessment of the intensity of unpleasant odours, two basic approaches can be distinguished: the analytical and sensory ones. Sensory techniques, including most frequently applied dynamic olfactometry, allow determination of odour concentration (of single substance, defined odourant mixtures, non-defined odourant mixtures) of the substances present in investigated samples, odour intensity and its hedonic quality. In the case of these techniques, the human nose plays the role of the “measurement sensor” (Suffet and Rosenfeld 2007; Munoz et al. 2010; Capelli et al. 2010; Guillot et al. 2012).

However, if a slight change in the emission of a single odourous substance has an enormous influence on the total emission resulting from the activity of a specific facility, e.g. sewage treatment plant, the analytical techniques become more advantageous than the sensory methods, as they allow for determining concentrations of individual components of the malodourous mixture ( Schwarzenbach et al. 2003; Nagaraj and Sattler 2005).

These techniques are mostly used for the identification and quantitative analysis of malodourous chemical compounds emitted into the environment. They are based on the characterisation of a given sample by accurate determination of its chemical composition. (Brattoli et al. 2011). They are distinguished by high repeatability, objectivity and accuracy (Zarra et al. 2009).

**Fig. 3:**
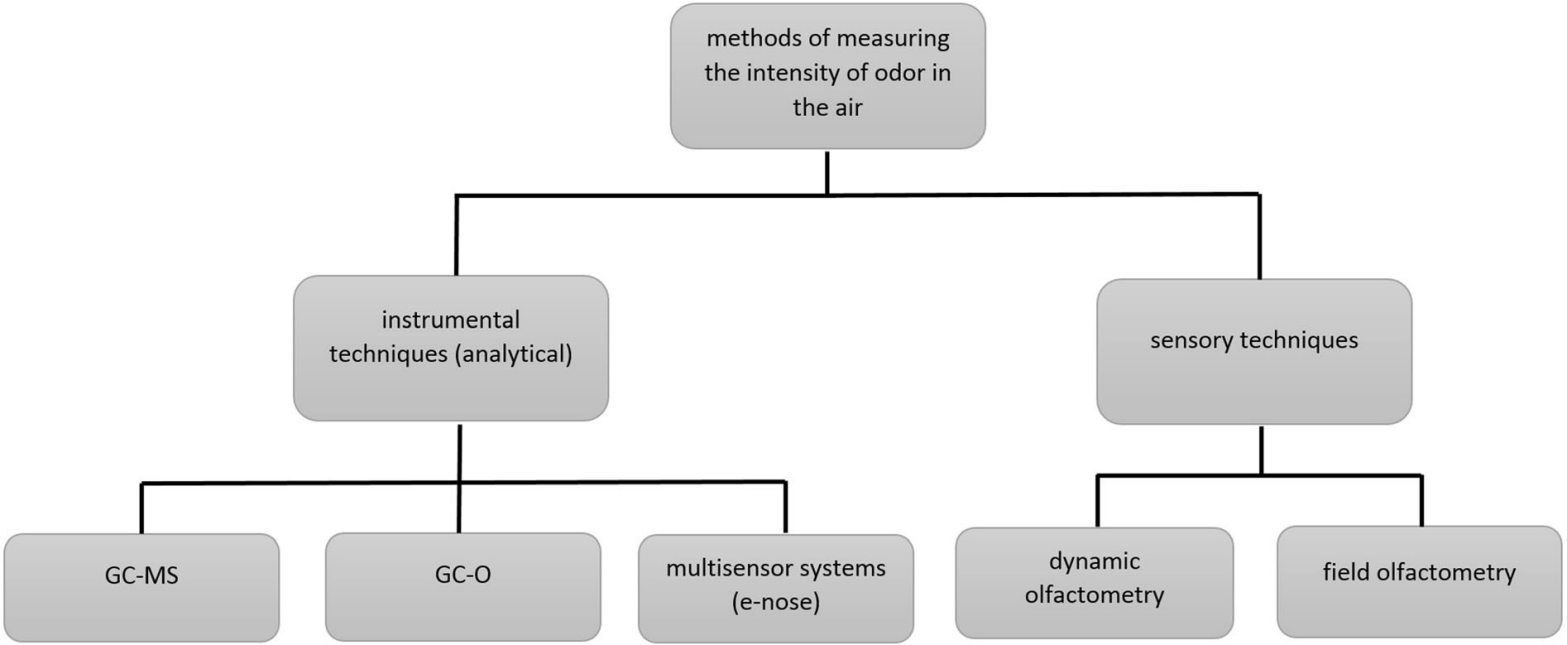
Classification of techniques for air quality assessment in terms of odour intensity

Figure 3 presents a diagram with a classification of the most important techniques, which are used to assess air quality in terms of odour intensity.

### Gas chromatography with olfactometric detection (GC-O)

GC-O allows for sensory assessment of compounds, which are released from the chromatographic column, together with an eluent stream. The human nose plays the role of an additional detector. Therefore, it is necessary to have a team of persons assessing the smell, just like in dynamic olfactometry. For each substance present in the analysed mixture, it is possible to perform quantitative and qualitative analysis simultaneously, i.e. stating whether a given compound can be sensorically detected at a strictly specified concentration, specifying what odour it has and determining the sensory intensity and the time of olfactory activity (Ferreira et al. 2002; Boudhrioua et al. 2003; Ferrari et al. 2004; Frank et al. 2004; Kleeberg et al. 2005; Bulliner et al. 2006; Zhang et al. 2010).

**Fig. 4:**
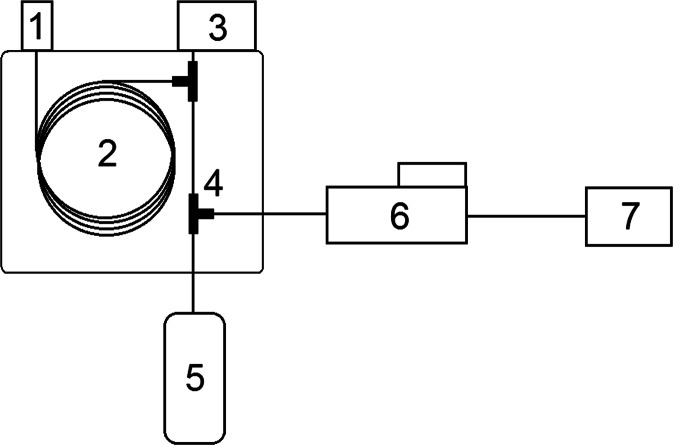
The design of the gas chromatograph with olfactometric detection. *1* dispenser, *2* chromatographic column, *3* detector, *4* stream divider, *5* humid air, *6* olfactometric detector, *7* signal generator

Figure 4 presents a diagram of a gas chromatograph design combined with an olfactometry detector. It is possible to assess the olfactory intensity of compounds eluted from the chromatography column, thanks to the presence of a specially constructed attachment, the so-called olfactometry stub nozzle, which fulfils the role of an additional detector (apart from it, the instruments also include a detector typical of gas chromatography—this is usually a flame ionisation detector or mass spectrometry detector). The eluate stream leaving the column is divided into two separate ones to make it possible for them to reach both detectors, which allows comparison of the obtained signals. This technique can be used to identify individual components of many complex odourous mixtures. The presence of, for example, a flame ionisation detector (FID), which is commonly used for GC, makes it possible to perform also qualitative and quantitative analyses of the determined compounds, in addition to GC (Plutowska and Wardencki 2008).

### Gas chromatography coupled with mass spectrometer (GC-MS)

One of the instrumental techniques for sensory analysis is certainly GC-MS. It is more and more commonly used in air pollution tests (Davoli et al. 2003; Dincer et al. 2006). Its use is advantageous especially for the determination of volatile organic compounds (VOCs) and volatile sulphur compounds, which constitute an important group of odours emitted from the sewage treatment plant (Zarra et al. 2008b, 2009). This technique also works very well in the case of identification of single odourous compounds present in very complex gas mixtures (Su et al. 2008; Pandey and Kim 2009; Woolfenden 2010a, b). For this reason, it gains increasing popularity in the air tests focusing on the assessment of olfactory quality, which is influenced by various manifestations of human activity, such as landfills or municipal sewage treatment plants (Defoer et al. 2002; Cadena et al. 2009).

Basic limitations of this technique are connected with too low concentration of some compounds present in odourous mixtures—it can often be lower than the limit of detection, which makes their analysis impossible (Staley et al. 2006). In recent years, the combination of the GC-MS technique with olfactometry detection (GC-MS-O) has been observed. Such a combination also allows more accurate determination of olfactory properties of individual compounds contained in mixtures (van Ruth 2001; Lo et al. 2008). That combination also makes it possible to better understand a correlation between the results of quantitative and qualitative analysis and olfactory properties of individual odourants. This approach is more and more broadly used in environmental tests, amongst other things (Brattoli et al. 2014).

### Electronic nose (e-nose)

An electronic nose is a measuring device, which is used for the sensory assessment of many chemical compounds from various sources. This device works in a manner similar to the human sense of smell. Odourous compounds are usually detected in it owing to the presence of a set of non-specific chemical sensors. However, its possibilities are much smaller than those of its “biological counterpart”, for example, due to the necessity of using a complex mathematical apparatus, which is responsible for proper interpretation of results (Rock et al. 2008; Wilson and Baietto 2009; Sankaran et al. 2012; Gebicki et al. 2014a, b; Boeker 2014).

**Fig. 5:**
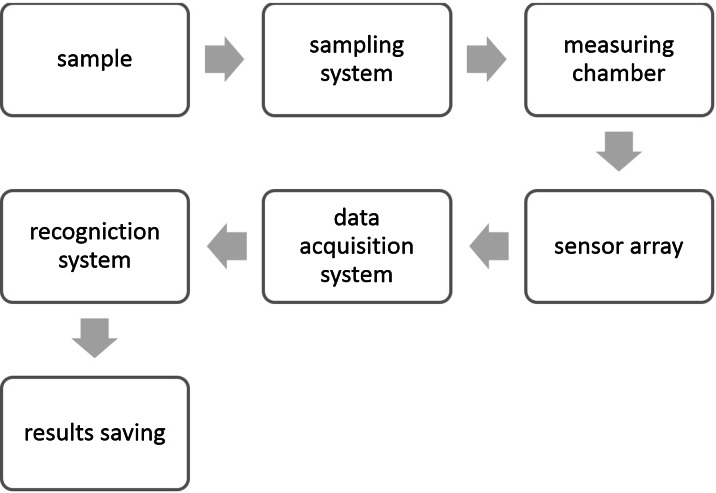
Diagram of e-nose operation

Figure 5 presents a diagram with the principle of electronic nose operation. The most important components of the relevant measurement system include:

- A sample collection system
- A system of sensors
- Data collection system
- Recognition system

A design of the sample collection system is aimed at ensuring the possibility of eliminating all undesirable factors, which can influence the response of the detector (Dymerski et al. 2011). The majority of currently available devices have two chambers in this system: the sensor chamber and the sample chamber. They are monitored during analyses in terms of temperature and humidity level (Hodgins 1995; Patel 2014). The sensor system makes it possible to measure desired properties—with variable selectivity. The data collection system is responsible for processing the data obtained during measurements and recording them in an appropriate form, whilst the recognition system is responsible for qualitative identification of odourous substances on the basis of comparing the olfactory profile of the sample with the reference profile in the database (Boholt et al. 2005; Micone and Guy 2007; Delgado-Rodríguez et al. 2012). The electronic noses allow for conducting “continuous” field tests as they do not exhibit, as opposed to the human nose, olfactory adaptation (Nicolas and Romain 2004; Giuliani et al. 2012); therefore, they are more and more often used for the assessment of environmental pollution. In this way, it is also possible to register small changes in concentration of a given substance in the tested gas medium (Romain et al. 2005; Szczurek and Maciejewska 2005; Sohn et al. 2006; Bootsma et al. 2014).

### Dynamic olfactometry

**Fig. 6:**
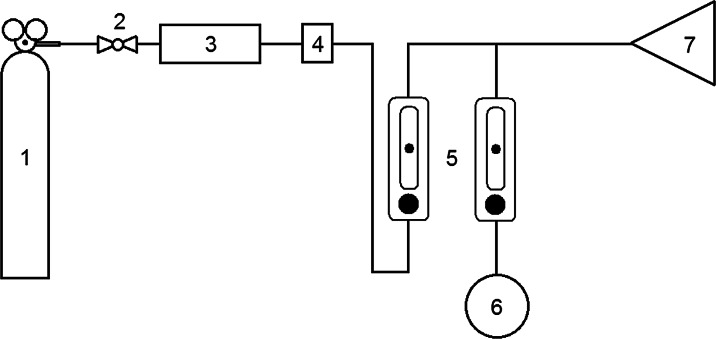
Diagram of a station for measuring olfactory intensity using the dynamic olfactometry technique. *1* cylinder with odourless air, *2* valve, *3* filter, *4* microfilter, *5* rotameters, *6* gas sample under analysis, *7* smelling mask

Dynamic olfactometry is a standardised measurement technique. It is preferred and, at the same time, most often used in the E.U. countries to determine concentration of individual odourants in mixtures of odourous substances emitted into the environment from various sources, also including municipal sewage treatment plants (Sironi et al. 2010; Belgiorno et al. 2012). Figure 6 presents a diagram of a research station design used for olfactometric measurements. These analyses are based on mixing a gas sample containing olfactory compounds with odourless neutral gas at specific ratios. The most frequently used olfactometers are equipped with four stations, which allows simultaneous presentation of a series of dilutions to more than one person. A panel of persons assessing the odour of the tested sample takes part in olfactometric tests. Such persons must receive prior training in terms of sensing even slight changes in olfactory intensity. Requirements for the persons participating in that type of research are contained in the standard EN 13725:2003 “Air quality. Determination of odour concentration by dynamic olfactometry.” The standard also contains, amongst other things, the procedure for odour determination using dynamic olfactometry, the sample collection techniques and the information about the method of obtained data recording and interpretation.

The results of measurements obtained using the dynamic olfactometry method are expressed in the European Odour Unit [1 ou_E_/m^3^]. This unit is defined as the quantity of the odourant or odourants, which after evaporation to neutral gas of 1 m^3^ volume under normal conditions induces a physiological reaction in the members of the assessment team, which is equivalent to that resulting from the European Reference Odour Mass (EROM) odour reference, which has also been evaporated to 1 m^3^ of neutral gas under standard conditions. Standard conditions, which are referred to in the definition above, are consistent with the ISO 10 780, i.e.:

- Room temperature—298.15 K
- Atmospheric pressure—101,325 Pa

Determination of odourant concentrations using dynamic olfactometry very often involves high costs connected with the preparation of a specialist olfactometric laboratory and ensuring that people who take part in tests have an olfactory sensitivity monitored on a regular basis.

The results obtained from this technique, especially for low concentrations of individual odourants, can considerably diverge from actual ones due to the possibility of changing the chemical composition of the sample between the time of its collection and the time of the analysis. Therefore, these tests are mostly recommended for determining olfactory compounds with high concentrations, much higher than emission ones, which occur in atmospheric air.

In many E.U. countries, the attempts have been made to introduce legal acts regulating olfactory pollution emissions. They define, amongst other things, admissible emissions of odourant concentrations in the environment and the procedure for measuring them. Due to changing conditions of urban infrastructure and the related increase in pollutants concentrations, it is necessary to update and further develop the recommendations contained in relevant legal acts, which takes place in many countries, both in Europe and in the world. The related procedures are time-consuming but, despite this, an increasing progress is observed in the legislation concerning environmental protection. Table 2 summarises the information about the most important legal regulations concerning prevention of olfactory onerousness.

**Table 2:** Selected legal acts in force in various countries, which concern the prevention of olfactory onerousness

Country	Legal act
Netherlands	2000—Nederlandse Emissie Richtlijnen NeR, Nederlandse Emissie-richtlijn Lucht §3.6 (NeR): Handleiding geur: Bepalen van het aanvaardbare hinderniveau van industrie en bedrijven (niet ve-ehouderijen)
Germany	1986—VDI 3881 Olfactometry: Odour Threshold Determination
	1994—Feststellung und Beurteilung von Geruchsimmissionen–Geruchsimmssions–Richtlinie (GIRL)
	2008—Feststellung und Beurteilung von Geruchsimmissionen–Geruchsimmssions–Richtlinie (GIRL)
Great Britain	1990—Environmental Protection Act—EPA
	2003—Technical Guidance Note IPPC H4 Horizontal Guidance for Odour
Japan	1972—The Offensive Odor Control Law in Japan
Denmark	2008—Dutch Legislation on Ammonia and Odour
North Korea	2005—New Odour Prevention Act

### Field olfactometry (FO)

The tools used for the assessment of odourous substances released into the environment include field olfactometers, which allow for in situ testing of concentration of odourous substances and odour emissions. These devices are portable so test results can be obtained in real time; the team of assessing persons defines the odour of substances in analysed samples. Field test results make it possible to identify sources of odourants and also to estimate the total odour emissions at a given measurement point. Environmental olfactometry is also used for the assessment of the degree of onerousness, the frequency of occurrence and the influence of odours on the comfort of living of inhabitants in a given area. The use of olfactometric techniques is connected with the necessity of having an experienced team of assessing persons, whose sensory sensitivity can be reduced due to various factors (Both et al. 2004).

**Fig. 7:**
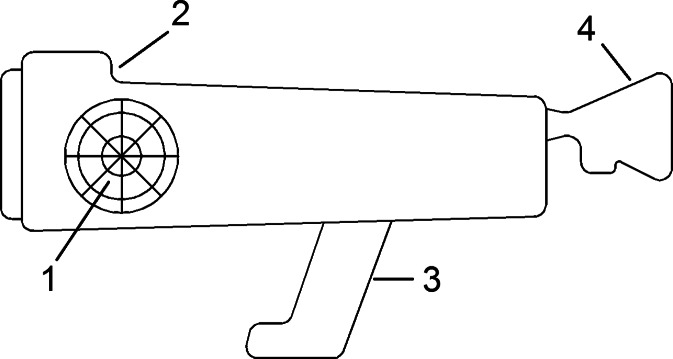
Diagram of field olfactometer design. *1* activated carbon filter, *2* electronic display of measurement results, *3* handle, *4* replaceable mouth and nose mask

The diagram of the field olfactometer design is presented in Fig. 7. This type of device is a kind of a gas mask equipped with a filter with activated carbon. At the beginning, the assessing person inhales clean air, purified on a filter, and, after a minute, gradually increases the share of the air collected from the environment and bypassing the activated carbon filter. The aim of the tests is determination of the numerical value of the dilution-to-threshold (*D*/*T*) ratio parameter, at which the odour of the gas mixture can be smelled by the assessing person.

However, no technical parameters have been defined for the research conducted using field olfactometry. No quality parameters or criteria concerning accuracy and precision have been determined. The results obtained can be considerably influenced by errors connected with variable perception or faulty operation of activated carbon filters. However, FO is more and more commonly used in the assessment of odour onerousness, which results from the activity of various facilities, including municipal engineering plants (Smeets et al. 2007; Capelli et al. 2011).

Table 3 presents the information, which can constitute the basis for a critical comparison of the measurement techniques used for the assessment of atmospheric air quality in terms of olfactory intensity (Nagy 1991; Hobbs et al. 1995; Di Francesco et al. 2001; Arshak et al. 2004; Rosenfeld et al. 2004; Bulliner et al. 2006; Sohn et al. 2008; Munoz et al. 2010; Zhang et al. 2010; Sówka 2010; Brattoli et al. 2011; Sówka et al. 2011; Le et al. 2013; Vergara et al. 2013; Dymerski et al. 2014).

**Table 3:** Comparison of measurement techniques used for the assessment of atmospheric air quality in terms of odour intensity

Method of analysis	Method of analysis	Advantages	Disadvantages
Analytical methods (e.g. GC-MS)	Samples collected at emission sources, next analysed using various detectors, e.g. MS	Possibility of accurate assessment of the content of individual analytes in the mixture, significant from the legislative perspective. Owing to high resolution, it is possible to identify the origin of compounds, and it is helpful when several emission sources occur Sample concentration is sometimes required	Sensory assessment of a sample is not possible, and sample representativeness and integrity depend on many factors, e.g. the type of container, collection time etc. The majority of methods does not allow for gas analysis at the human nose level Expensive and labour intensive
GC-MS-O	As above, except for the fact that a half of the flowing sample after separation is directed to a person in the sensory panel	Additional significant human factor Cross-tables with results between the MS detector and olfactometry can provide new information about the contribution of single odourants to the odour of the entire mixture	Due to the separation of analytes in the GC system, odourous substances cannot be analysed together, potential synergistic or antagonistic effects cannot be assessed High dependence between the composition and the representativeness and integrity of the sample
Electronic noses	Devices consisting of a matrix of sensors and an appropriate data processing system	Identification of odour markers at a lower level than in some analytical methods, e.g. using photoionization detection Possibility to skip the sample collection stage, on-site measurement Possibility of measuring odours at places, which are difficult to reach	Identification of odours at a lower level than in olfactometry, which influences the ability to assess the impact of the odour Selected sensors are sensitive only to specific substances, other unknown substances are no longer identified Complicated calibration, necessity for training, e.g. by correlation with olfactometric results to assess the offensive quality of odours
Olfactometric techniques	Main methods of measurement using sensory panels of field olfactometers, e.g. Nasal ranger, odour detection and assessment is based on the sense of smell.	Elimination of the problem of sample representativeness and integrity in the case of field olfactometry Useful in the assessment of olfactory onerousness	Various factors influencing odour assessment by panel members Training is required to ensure the objectivity of panel members, high costs Differences in odour assessment by sensory panel members Underestimation of olfactory onerousness for the community

#### Exemplary information about the measurement techniques used in the assessment of atmospheric air quality in terms of odour intensity in a sewage treatment plant

Table 4 presents the information about the measurement techniques used for the assessment of atmospheric air quality in terms of odour intensity.

**Table 4:** Measurement techniques used for the assessment of atmospheric air quality in terms of odour intensity

Determined substances	Measurement technique used	References
Volatile organic compounds	Electronic nose	Stuetz et al. (1999a)
		Bourgeois and Stuetz (2000)
		Dewettinck et al. (2001)
		Bourgeois and Stuetz (2002)
		Bourgeois et al. (2003a)
		Bourgeois et al. (2003b)
		Onkal-Engin et al. (2005)
		Guz et al. (2015)
	Electronic nose, olfactometry	Stuetz et al. (1998)
		Littarru (2007)
		Capelli et al. (2008)
	GC-MS	Escalas et al. (2003)
		Wu et al. (2006)
		Zarra et al. (2008a)
	Olfactometry	Gostelow et al. (2001)
		Suffet et al. (2004)
		Suffet and Rosenfeld (2007)
		Capelli et al. (2009)
	Electronic nose, GC-MS, olfactometry	Zarra et al. (2014)
H_2_S	Electronic nose, olfactometry	Stuetz et al. (1999b)
	Olfactometry	Dincer and Muezzinoglu (2007)
CH_3_SH	Electronic nose	Nake et al. (2005)
Organic sulphur compounds	GC-MS	Cheng et al. (2007)
		Ras et al. (2008)
		Sheng et al. (2008)
	Olfactometry, GC-MS	Rajbansi et al. (2014)
Volatile organic compounds and organic sulphur compounds	GC-MS	Godayol et al. (2011)
	GC-MS-O	Ranau et al. (2005)
	GC-MS, olfactometry	Zarra et al. (2008b)
		Zarra et al. (2009)
	Olfactometry	Barczak et al. (2012)
		Naddeo et al. (2012)
		Zarra et al. (2012)
		Almarcha et al. (2014)
		Baltrenas et al. (2013)
Sulphur and nitrogen odourants	GC-MS	Turkmen et al. (2004)
Sulphur, nitrogen odourants and volatile organic compounds	GC-MS, GC-O	Agus et al. (2012)

#### Trends in development of analysis and monitoring of malodourous substances in the air

Legislative bodies in the majority of developed countries undertake the problem of regulating admissible olfactory pollutant emission (Henshaw et al. 2006; Nicell 2009; Bokowa 2010). In many countries, the regulations pertaining to odour emissions are continuously changed and improved. The electronic noses are beginning to be accepted as the devices used in reference methodologies to measure odours (Engg and College 2013). For example, in France (Barriada-Pereira et al. 2010), new regulations make it possible to use the e-noses to monitor odours from sewage waste neutralisation processes (rendering) and composting. On-line monitoring is possible with the use of one or several e-noses at the distances regulated by the act. Also, the frequency of measurements performed with the electronic nose is specified by the act. Due to the specificity of electronic nose operation, these devices can successfully complement current odour measurement techniques and such improvements are treated very seriously in many countries in the world. The effectiveness of introduced legal solutions is confirmed by decreasing number of complaints, which means that the impact of odour-related onerousness on communities inhabiting the areas around the main emitters is reduced (Loriato et al. 2012).

The electronic nose can be used as a device complementing other analytical techniques, namely sensory analysis techniques. At present, the devices allowing for analysing odours and controlling admissible concentrations of malodourous substances are available on the market. Moreover, work on improvement of these devices is in progress and new better versions of their prototypes are developed. One of the basic problems related to sensors is their stability during temperature and humidity changes as well as sensor response drift in time. In recently published studies, Dentoni et al. (2012) describe an innovative electronic nose developed for monitoring of the environment, which includes solutions for signal drift compensation and for sample humidity regulation. Challenges concerning the use of electronic noses in environmental monitoring should not focus only on the development of new sensors or data processing methods, but they should rather concentrate on the adaptation of existing devices to external applications. In the years to come, possibilities of using the electronic noses under real conditions as portable devices for controlling outdoor air should become more popular (Capelli et al. 2013).

The most critical aspect limiting the use of electronic noses is the lack of specific regulations for their standardisation. As mentioned above, the electronic noses for environmental monitoring are complex devices and the way, in which they are used, is connected with high diversity, especially as regards the training and data processing stages. The actual standardisation of the devices in terms of their proper use and objectification of tests performed, which is aimed at simplification and unification of the stages of analysis, can contribute to popularisation of such an instrument for environmental applications in the future. One of the first attempts at the standardisation of outdoor air measurements using the electronic nose was presented by a Dutch standardisation institute in the form of the NTA-9055 document.

### Summary

Due to the issue of odour onerousness, which has not been regulated yet in many countries in the world, a basic step for this purpose includes objectification of the assessment of odour impact and odour limits. Individual odours can cause a broad spectrum of sensations, and the influence of an odour on odour onerousness may result from a few of its characteristic features defined by the FIDOL acronym (Loriato et al. 2012):

- Frequency of the odour occurrence
- Intensity
- Duration of the exposure
- Offensiveness of the odour, subjective
- Location of the odour

One of main problems connected with the smell is the issue of the person’s exposure time to unpleasant odours, which does not cause onerousness. It can be assumed that there exist two concepts, which allow for minimising this problem—limiting the odour emission level so that it is not detected all the time or releasing odours periodically at higher concentration on a short-term basis, owing to which the exposure can be reduced. To prevent air quality deterioration, emission odour standards have been determined in many countries in the world, which use:

- The odour unit allowing for defining the concentration of individual odourants or their mixture in a specific manner (*c*_od_ [ou_E_/m^3^])
- Standards pertaining to odour emissions (*q*_od_ [ou/h])
- Diagrams of minimal distances of sources of emissions from residential buildings
- Analysis of complaints concerning odours

In the assessment of the degree of the intensity of the impact of unpleasant smells, the odour unit is most often used, which is defined as the quantity of the odourant and/or odourants, which induces a physiological reaction of the assessing team (odour threshold value) after evaporating a cubic metre of gas under standard conditions by a representative group of people, the so-called sensory panel. A popular method of assessing the degree of odour impact is the use of mathematical models to predict the level of odour dispersion after taking into account the wind rose, size of emission source, terrain topography and meteorological data. Owing to the use of the dilution of an atmospheric air sample to its odour threshold value (*D*/*T* counterpart of *c*_OD_), it is possible to perform quantitative and qualitative analysis of odours or their mixtures in accordance with the methods contained in relevant standards (e.g. ASTM Method E679-04 or European Method EN 13725:2003).

A lot of classical methods for determining malodourous substances are considered to be too expensive to create commonly available measuring instruments or develop environmental monitoring systems. High hopes for the development of such systems are placed on sensor techniques. Odour analysis is more and more frequently performed with the use of electronic noses. Despite the low applicability of these devices for the monitoring of air pollution as compared with olfactometry, their application is growing. The stages of selecting appropriate sensors and data processing method make it possible to use electronic noses for the analysis of odours of various origins. One of the main advantages of these devices is the possibility of skipping the first stage of sample preparation for the analysis and the possibility of their field use as a portable unit to monitor emission sources in real time. Numerous literature reports, which are devoted to theoretical fundamentals of electronic nose operation and practical possibilities of their use, are the basis for concluding that the development of odour analysis is connected with the use of electronic noses. The authors of the article hope that the reference methods in the analysis of malodourous substances will soon be extended in many countries to include the possibility of using the electronic nose. Such devices would be an element of early warning against sudden odour hazards. For short-term odour onerousness, it will be possible to register them using the e-nose, which, in turn, is often not possible after qualified personnel arrives on site to perform field olfactometry measurements. Furthermore, there are situations in which the performance of field olfactometry measurements might constitute a health hazard for people.
